# Measuring guide-tree dependency of inferred gaps in progressive aligners

**DOI:** 10.1093/bioinformatics/btt095

**Published:** 2013-02-23

**Authors:** Salvador Capella-Gutiérrez, Toni Gabaldón

**Affiliations:** ^1^Bioinformatics and Genomics Programme, Centre for Genomic Regulation (CRG), Doctor Aiguader 88 and ^2^Universitat Pompeu Fabra (UPF), 08003 Barcelona, Spain

## Abstract

**Motivation:** Multiple sequence alignments are generally reconstructed using a progressive approach that follows a guide-tree. During this process, gaps are introduced at a cost to maximize residue pairing, but it is unclear whether inferred gaps reflect actual past events of sequence insertions or deletions. It has been found that patterns of inferred gaps in alignments contain information towards the true phylogeny, but it is as yet unknown whether gaps are simply reflecting information that was already present in the guide-tree.

**Results:** We here develop a framework to disentangle the phylogenetic signal carried by gaps from that which is already present in the guide-tree. Our results indicate that most gaps are incorrectly inserted in patterns that, nevertheless, follow the guide-tree. Thus, most gap patterns in current alignments are not informative *per se*. This affects different programs to various degrees, PRANK being the most sensitive to the guide-tree.

**Contact:** tgabaldon@crg.es

**Supplementary information:** Supplementary data are available at *Bioinformatics* online.

## 1 INTRODUCTION

Multiple sequence alignments (MSA) play a central role in modern molecular biology, and they are used in a broad set of applications, ranging from phylogenetic analyses to the identification of functional motifs (Notredame, 2007). As the quality of an alignment will inevitably affect the quality of downstream analyses, different strategies have been proposed to improve the quality of MSA. In the context of the reconstruction of phylogenetic trees to establish the evolutionary relationship among a given set of sequences, a major problem is how to extract phylogenetic information from inferred gaps. Theoretically, inferred gaps in an alignment should represent past events of sequence insertions or deletions. When this is the case, a proper incorporation of gap information into phylogenetic inference has been shown to be informative (Dwivedi and Gadagkar, 2009). In practice, however, gaps are generally introduced, at a penalty cost, to maximize residue pairing scores. Most alignment reconstruction programs use a progressive approach in which most similar sequences are aligned first, following a guide-tree. During the alignment reconstruction, optimization is based on two main components: residue pairing and gap penalties. In contrast to residue pairings, where empirical models exist, gap penalties are generally rather arbitrary, with some notable exceptions (Wrabl and Grishin, 2004). It is unclear to which degree inferred gaps in alignments correspond real past events of insertions and deletions. As a result, highly gapped regions are commonly considered unreliable (Golubchik *et al.*, 2007), and it is common practice to ignore them before phylogenetic analyses (Capella-Gutierrez *et al.*, 2009; Talavera and Castresana, 2007). A recent study has reported an unexpected accuracy of maximum parsimony trees reconstructed solely from the information contained in presence/absence patterns of inferred gaps in protein alignments (Dessimoz and Gil, 2010). This result has been attributed to phylogenetic information contained directly in gaps introduced by alignment programs, and it would imply that current phylogenetic methods could be improved by exploiting such information. However, for this to be true, gaps should carry independent phylogenetic information, truly reflecting past evolutionary events, such as insertions and deletions. Alternatively, because of the progressive nature of the alignment reconstruction, gap patterns may simply reflect information already present in the guide-tree, which is usually reconstructed from pairwise sequence distance information. If this would be the case, usage of the gap patterns in phylogenetic reconstruction would be biased towards the guide-tree, which is prone to contain errors. Disentangling the two scenarios is of central importance to improve the existing multiple sequence alignment methods and to design proper strategies to exploit the potential information contained in gaps. At the same time, this task is challenging, given the lack of a proper framework to measure the effect that guide-trees have in the introduction of gaps. Here, we develop a novel approach to assess whether the information contained in gap patterns reflect true evolutionary events, and whether this is different from the phylogenetic signal already present in the guide-tree. We apply such framework to several synthetic and real datasets and using five different alignment strategies that represent the main alignment approaches (Notredame, 2007). Our results show that most gaps are incorrectly inserted in patterns that, nevertheless, tend to follow the guide-tree. Hence, gaps carry little additional information, distinct from that already present in the guide-tree. Our results emphasize the role of the guide-tree when alignments of gappy data are used for evolutionary analyses. Although the impact of this effect varies across datasets, some alignment algorithms are consistently more affected than others. In all cases, the errors could be reduced by either using the known true tree as a guide or—when this is not available—applying an iterative co-estimation method that infers both the tree and the alignment.

## 2 METHODS

### 2.1 Simulated sequence datasets

As a synthetic scenario in which the real history of insertion and deletion events is known, we worked with one of the simulated dataset previously used for the benchmarking of trimming methods (Capella-Gutierrez *et al.*, 2009). This consists of 600 sets of 32 simulated protein sequences each divided into two categories, asymmetric and symmetric, depending on the original tree topology used to simulate the alignments.

### 2.2 Real sequence datasets

We used two different sets of real sequences. First, the original data from Dessimoz and Gil (2010), which was used to show phylogenetic information in gaps, was accessed from their website (www.cbrg.ethz.ch/research/msa). This dataset contains groups of orthologous proteins for three different taxonomic clades eukaryotes (609 orthologous groups), fungi (844) and bacteria (1999). In addition, we downloaded the original data from Marcet-Houben and Gabaldón (2009), accessed through the public database www.phylomedb.org (Huerta-Cepas *et al.*, 2011). This dataset (phylome ID = 7), which we will refer to as *yeast*, contains trees for all *Saccharomyces cerevisiae* proteins across a phylogeny of 12 *Saccharomycotina species*. The data were filtered out to keep only 857 sets of one-to-one orthologous proteins.

### 2.3 Alignment programs

We reconstruct MSAs using five different approaches, which could be classified depending on the scoring strategies into *scoring-matrix-based* MAFFT FFT-NS-2 or FFT-NS-1 v6.712b depending whether an input guide-tree is used (Katoh and Toh, 2008) and ClustalW2 v2.0.12 (Larkin *et al.*, 2007), *consistency-based* MAFFT L-INS-i v6.712b and T-Coffee v9.01 (Notredame *et al.*, 2000); and *tree-aware-gap-placing* Prank v.100701 (Loytynoja and Goldman, 2008). All programs were used with default parameters. Additionally, SATé II (Liu *et al.*, 2012), a program which combines the estimation of the MSA and the maximum-likelihood (ML) phylogenetic tree, was used to evaluate its performance as an alternative to the rest of aligners used in this work. MAFFT with auto option and Prank +F were used as engines in SATé II.

### 2.4 Accuracy and precision of gap placement

Using the true alignments from the simulated datasets, we compared the opening positions of gaps in reconstructed alignments. Gap positions were recoded using the corresponding surrounding residues in reference and reconstructed alignments (Supplementary Fig. S1). Gaps opened between the same residues in the reference and the test alignment were considered true positives (TP), whereas those present only in the reference or in the test alignment were considered as false negatives (FN) and false positives (FP), respectively. Finally, true negative (TN) represents residues well-placed regarding to the number of gap-blocks opened before each residue. Precision was computed as P(aligner) = TP/(TP + FP), and accuracy was computed as A(aligner) = (TP + TN)/(TP + FP + TN + FN).

### 2.5 Tree discordance tests

Reconstructed trees were compared in terms of their normalized split-distance (Robinson and Foulds, 1981) with a canonical or a wrong tree. The canonical tree was the real tree in the simulated dataset and the canonical species tree for the Dessimoz and Marcet-Houben datasets (these trees are represented in Supplementary Fig. S2). The ‘wrong tree’ is an alternative topology, which has the highest distance in terms of wrong splits (100%) to the canonical species tree. As there are many possible wrong trees with the maximal distance to the canonical tree, for one of the datasets (simulated data symmetric topology) we repeated the same procedure using 100 alternative possible wrong trees, and the results obtained were similar (Supplementary Fig. S3), and thus a single wrong tree was used in subsequent analyses. The wrong trees used for the different datasets are provided in Supplementary Fig. S4). The ETE package (Huerta-Cepas *et al.*, 2010) was used to perform all operations related to phylogenetic trees.

### 2.6 Gap parsimony reconstruction

To assess the amount of phylogenetic information contained in gap patterns, we used the procedure proposed by Dessimoz and Gil (2010). That is, alignments are re-coded in presence/absence patterns of gaps (two-state character: for a given alignment, each column containing at least one gap was considered a character and the presence/absence of a gap its state). Subsequently, a maximum parsimony tree is reconstructed using the gap patterns from the recoded alignment (GP), using Wagner parsimony as implemented in Darwin v2.0 (Gonnet *et al.*, 2000) and as described in Dessimoz and Gil (2010).

### 2.7 Maximum-likelihood phylogenetic reconstruction

ML phylogenetic trees were reconstructed using PhyML v3 (Guindon *et al.*, 2010) with a discrete Γ-distribution model with four rate categories plus invariant positions, estimating the α-parameter and the fraction of invariant positions from the data. LG was used as evolutionary model, and branch and topology were optimized.

## 3 RESULTS

### 3.1 Most gaps in sequence alignments are incorrectly inserted

**Fig. 1.**
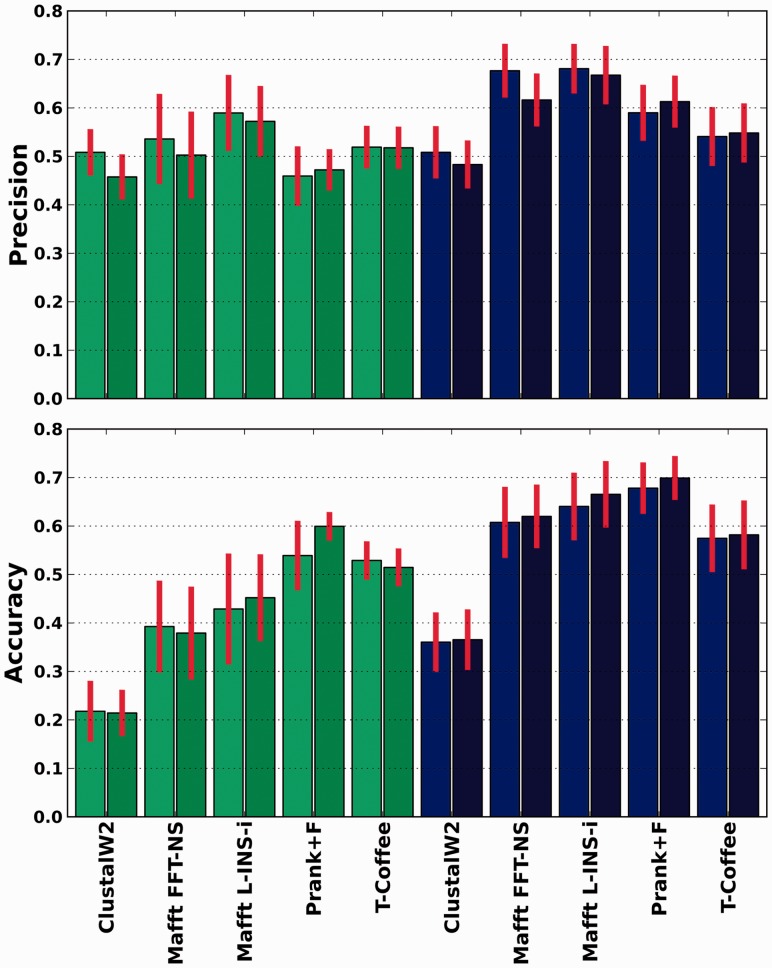
Precision and accuracy of gap placements for the different strategies used to reconstruct MSAs divided according to the nature of the simulated data (green bars: asymmetric; blue ones: symmetric) and when using the normal procedure (light bars) and when the use of the correct tree as a guide-tree is enforced (dark bars)

Accuracy of sequence alignments is generally assessed on the basis of residue pairings, but only recently developed distance measures that also include similarities in terms of gap placement have been developed (Blackburne and Whelan, 2012). However, these distances do include information from residue pairing differences, making it difficult to assess what is the relative distance in terms of gap positioning and residue pairings. To assess to what degree gaps were inserted at correct positions, we used a set of simulated sequences (Capella-Gutierrez *et al.*, 2009). Sequences in these sets were re-aligned, and the positions of the newly inserted gaps were compared with those in the reference alignments. Our results (Fig. 1) show that, in any given alignment, a significant fraction of the inserted gaps (30–80%) are placed at incorrect positions. This was true for all aligners, and even when the correct tree was used as a guide.

### 3.2 Gap patterns follow the guide-tree and carry little additional phylogenetic information

If most gaps are incorrectly placed, how can gap patterns carry phylogenetic information as suggested by recent reports (Dessimoz and Gil, 2010)? One possible explanation to this apparent conundrum is that gaps are placed following a pattern that is consistent with the phylogeny. Multiple sequence aligners use evolutionary information that is provided by the guide-tree, a cladogram that dictates in which order the sequences are initially aligned to each other. This guide-tree is generally built from the pairwise distances of the sequences involved; thus, it inherently carries phylogenetic information. To test the extent to which gap patterns follow the guide-tree, we measured the effect of altering the guide-tree. We tested this in the previously mentioned simulation dataset and in two real datasets that were used in Dessimoz and Gil (2010), which comprises alignments from bacteria, fungi and eukaryote sequences, and one taken from Marcet-Houben and Gabaldón (2009) comprising sequences from yeast species. More specifically, we repeated each alignment in the previously mentioned datasets by using (i) the normal procedure, enabling the program to build its own guide-tree, (ii) forcing the use of the correct tree (or a canonical species tree) as a guide-tree and (iii) forcing the use as a guide-tree of a synthetic ‘wrong’ tree having the maximum split-distance to the correct tree. Note that the use of the wrong tree is made with the sole purpose of maximizing the differences as to better visualize the effect of the guide-tree.

If gap patterns are mostly dictated by the guide-tree, then the use of a distinct guide-tree should have a large impact on the ability of gap patterns to reconstruct the correct tree. Indeed, under such conditions, one would expect that information contained in gaps is biased towards the guide-tree to a degree that would reflect the strength of the guide-tree dependency of the aligner. Maximum parsimony reconstruction from patterns of gap presence/absence has been used to show that gaps contain unexploited phylogenetic information (Dessimoz and Gil, 2010). We thus applied the same approach using the three different strategies aforementioned. As our procedure requires the program to enable using a user-defined guide-tree without altering it, we limited our analyses to ClustalW, T-Coffee, PRANK and MAFFT, using the latter in two different modes: the consistency-based L-INS-i and the progressive FFT-NS-1 (Katoh and Toh, 2008; Larkin *et al.*, 2007; Loytynoja and Goldman, 2008; Notredame *et al.*, 2000). Thus, although our choice of programs is limited, it covers a range of alignment strategies from progressive to iterative, going through consistency-based and phylogeny-aware strategies (Kemena and Notredame, 2009).

**Fig. 2.**
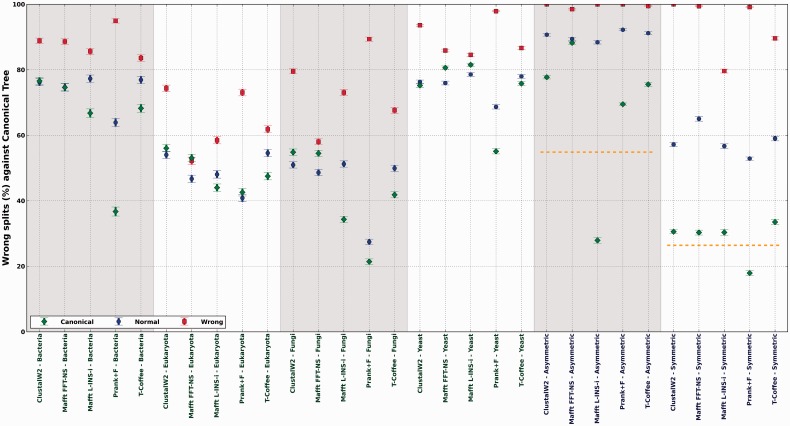
Mean distance, in term of wrong splits, to the canonical trees of the different gap parsimony trees reconstructed after allowing the programs to build its own guide-tree (blue dots) or forcing them to use either the canonical tree (green diamonds) or an alternative topology (red squares), with maximum split-distance to the canonical tree. Wrong splits measure the number of topological differences between two given trees. Yellow dashed lines in the simulated datasets indicate the signal retrieved from the real gaps using the same gap parsimony approach

Figure 2 shows the distance to the correct tree, of parsimony trees reconstructed from gap patterns (gap parsimony) in alignments using the alternative three guide-trees aforementioned. In most cases, the use of the wrong tree as a guide-tree destroyed most of the signal towards the true tree, indicating that wrong guide-trees mislead gap placement. Conversely, the use of the correct tree as a guide tends to improve the phylogenetic information contained in gaps. These results indicate, as expected, that guide-tree accuracy is an important factor determining the phylogenetic information contained in gaps. However, this does not solve the issue of whether gaps harbor additional information as compared with the guide-tree. Some additional lines of evidence suggest that gaps mostly carry information dictated by the guide-tree. First, alignments reconstructed from wrong guide-trees carry phylogenetic information pointing towards that wrong topology (Supplementary Fig. S5). Second, the guide-tree reconstructed by the alignment program is generally a better estimator of the true topology than the tree reconstructed from gap patterns (Supplementary Fig. S6), indicating that the use of gap parsimony recovers less phylogenetic information than that already contained in the guide-tree. Finally, gap parsimony trees were reconstructed for the simulated alignments without realigning them to evaluate whether these perfectly placed gaps are able to resemble the trees used to generate them. As it can be seen (Fig. 2 yellow dashed lines), simulated gaps cannot properly reconstruct the simulated phylogeny. Of note, the normal process of alignment reconstruction (blue dots) significantly erases the signal in gaps, and only in some cases, and always using the canonical tree as a guide (green diamonds), the recovered signal is similar to the one present in real gaps. The concatenation of gap patterns from multiple genes increased the signal towards the canonical tree only when the correct guide-tree was used, and, in some cases, the concatenation of 300 genes was not enough to fully-recover the canonical phylogeny (Supplementary Table S1). These results highlight the difficulty of the gap parsimony approach to recover sufficient phylogenetic information, even when the correct guide-tree is used.

### 3.3 A measure for guide-tree dependency

**Fig. 3.**
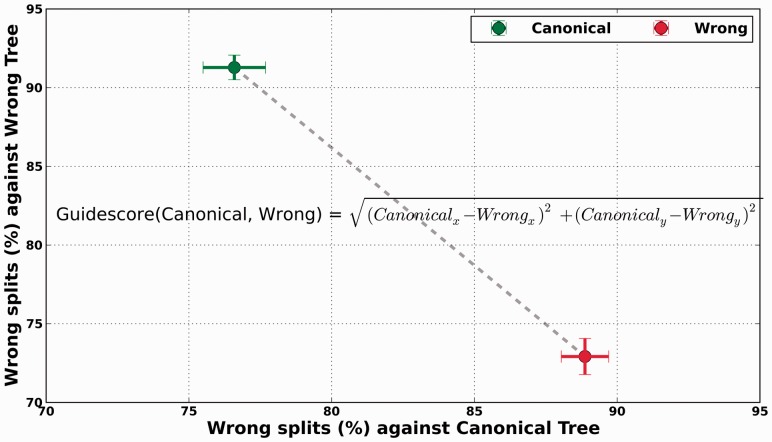
Example showing how to compute the *guidescore* for two alternative (sets of) trees computed using different approaches. In this case, the score is computed considering the gap parsimony trees inferred after forcing ClustalW2 to use the canonical topology and an alternative one with the maximum split-distance to the canonical tree

**Fig. 4.**
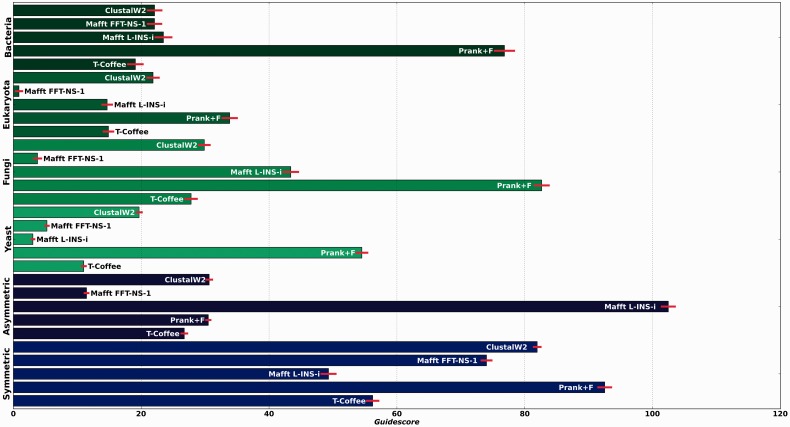
*Guidescores* computed for all available datasets, simulated data in blue and real data in green, for all approaches mentioned in the study. *Guidescores* were computed between trees inferred after forcing programs to use either the canonical reference trees or trees with maximum split distance to reference one

We have shown that most gaps are inserted incorrectly, but following a pattern mostly dictated by the guide-tree. This is even true for our simulated datasets when the correct tree is used as a guide. These effects seem to be present in all programs but to different degrees. A measure that would allow us to comparatively assess the guide-tree dependency of the different aligners in terms of their gap placement would be useful to make informed choices of methodologies or parameters. We here propose the following methodology to derive a simple measure that captures the effect of guide-tree: given a 2D space where the coordinates are, respectively, the split distances to (i) a canonical tree (the true tree) and to (ii) a wrong tree with maximum split-distance to the canonical tree, a given tree topology could be represented by its respective coordinates. If two alternative trees, each one derived from a different alignment using either the canonical tree or the wrong tree as a guide, are projected into this space. Then, the euclidean distance between these points will effectively measure the effect on the topology of altering the guide-tree. Such a plot and the derived distance are shown for the bacterial dataset and ClustalW2 (Fig. 3). In this framework, a high level of guide-tree dependency will produce trees that are close to the guide-tree, thus maximizing the distance in the mentioned space. We computed this value, which we will refer to as *guidescore*, for other combinations of aligners and datasets (Fig. 4). Our results indicate that the phylogeny-aware method PRANK is generally the most dependent.

Similar results are obtained when two random trees with maximal topological distances are used (Supplementary Fig. S7). Although maximal distances are good to better distinguish across more or less affected programs, more modest distances are perhaps more indicative of the expected impact in real cases where the reconstructed guide-tree is expected to contain a moderate number of wrong partitions. Results for the different methods and datasets, at varying degrees of topological distances used for the *guidescore* are shown in Supplementary Figures S8–S10.

**Fig. 5.**
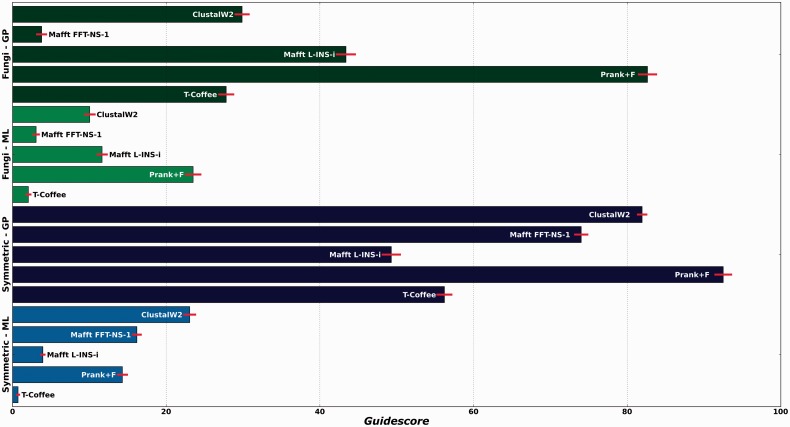
*Guidescores* for two datasets, one simulated (blue) and another one real (green), using all available methods and considering in this case two alternative approaches for reconstructing phylogenetic trees: gap parsimony (darker colors) and maximum-likelihood (lighter colors). *Guidescores* were computed between trees inferred after forcing programs to use either the canonical reference trees or trees with maximum split distance to the reference one

The *guidescore* measure can be applied to assess the effects of guide-trees on other reconstruction methods, and we here assessed the impact of guide-tree on maximum-likelihood reconstruction, using the same framework (Fig. 5). Our results indicate that guide-tree determination affects ML phylogenetic reconstruction to a much lower degree than gap parsimony, suggesting that gap patterns are more affected by guide-tree determination than residue pairings.

### 3.4 Strategies to overcome guide-tree dependency of gap placements

We finally set out to explore potential strategies that would serve to overcome the shown effect of gap tree dependency on gap placement. In particular, we explored two possible strategies (i) minimize the effect of guide-tree dependency and (ii) select gaps that are more likely to contain true phylogenetic information (use of consistency-based alignment trimming). We want to note that our intention is not to explore the full range of possibilities, but rather to show that the observed effect can be tackled. Intuitively, methods that iteratively reconstruct trees and alignments, such as that implemented in SATé (Liu *et al.*, 2012), should be less prone to the effect of an initially set guide-tree, as this will be changed through the iterations. Similarly, averaging over different aligners by means of consistency-based methods, such as M-Coffee (Wallace *et al.*, 2006), would be expected to minimize the effect. Indeed, as shown in Supplementary Figure S11, both strategies were found to be among the least affected by the guide-tree in most of the datasets. Of note, the reduction of the guide-tree dependency was also shown when SATé was run using PRANK as the underlying alignment program, indicating that an iterative approach, as expected, reduces guide-tree dependency even when highly dependent methods are used. However, despite reducing the dependency of the guide-tree, these approaches had little effect in the accuracy of the placement of gaps (Supplementary Fig. S12).

**Fig. 6.**
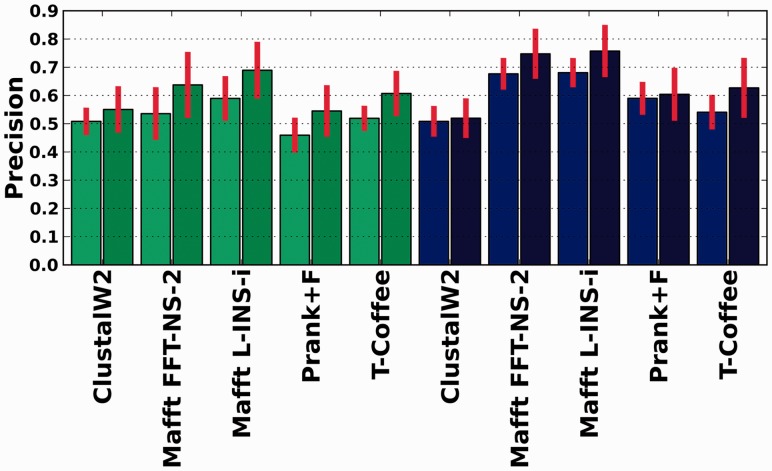
Accuracy of gap placements for the different strategies used to reconstruct MSAs before (light colors) and after (darker colors) trimming the alignments. Green bars correspond to asymmetric data and blue ones to symmetric simulated data

Finally, besides minimizing the effect, one may wish to select those gaps that are less likely to be the result of guide-tree guidance, and thus expected to contain independent phylogenetic signal. To do so, we investigated whether consistency-based trimming, as the one implemented in trimAl v1.3 (Capella-Gutierrez *et al.*, 2009), served to select gaps that are more likely to contain true phylogenetic information. To do so, we aligned each set of sequences in forward and reverse orientation [i.e. Head or Tails approach (Landan and Graur, 2007)] and then trimmed the alignment using trimAl with a cut-off of 0.05 consistency score. The rationale behind this approach is that by reversing the order of the sequences, one expects to alter more the insertion of gaps that are arbitrarily positioned (e.g. when there is a tie in the scores of two alternative gap placements) as compared with those that are consistently placed at the same position. Our results indicate that the precision of gaps present in trimmed alignments was significantly higher than in non-trimmed ones (Fig. 6). Note that other approaches such as using different aligners or varying gap penalties can also be used with the purpose of distinguishing between variable and robust gap placements.

## 4 DISCUSSION

Altogether our results indicate that most of the apparent phylogenetic signal carried by gaps in this analysis is actually a result of the preferential inclusion of shared gaps in sequences that are closer in the guide-tree. In other words, under these circumstances, many gaps do not contain additional phylogenetic information *per se* but rather reflect information already present in the guide-tree. Several lines of evidence support this. First, the initial guide-tree produced by the alignment program is highly similar to the canonical tree (Supplementary Fig. S6), indicating that it carries a strong phylogenetic signal. Importantly, this guide-tree is usually more similar to the canonical tree than the parsimony tree, solely based on gap information, indicating that the use of gaps in a parsimony framework as implemented here actually erases part of the signal contained in the guide-tree. Second, the use of a clearly wrong guide-tree to guide the process erodes the phylogenetic signal contained in gaps and biases it towards the wrong tree topology (Fig. 4 and Supplementary Fig. S5). Blackburne and Whelan (2012) already noted that the different placement of gaps by different aligners rarely altered the inferred evolutionary histories of insertions and deletions events, but failed to propose a possible source for such apparent contradiction. Our results provide an answer to this conundrum by showing that all aligners follow a similar guide-tree in different ways, thus resulting in disparate gap patterns that are nevertheless compatible with the same guide-tree. This is reinforced by our finding that, even using the same correct guide-tree, different alignments will place gaps in different patterns, compatible with the guide-tree, but mostly at positions that do not correspond with real sites of past insertions and deletion events. Hence, the recovery of correct phylogenetic information from gaps may simply indicate that an accurate guide-tree was used and not necessarily that gaps are correctly inserted following patterns of real past events of insertions and deletions.

We consider that these results are not in contradiction with the idea that insertions and deletions are rare evolutionary events that can be used for phylogenetic reconstruction. Indeed, we share the opinion of Dessimoz and Gil (2010) and others that an effort should be made in finding new ways of exploiting this information. Using a simulated framework where all gaps correspond to simulated indels and their true position is known, their usefulness for phylogenetic reconstruction has been established (Dwivedi and Gadagkar, 2009). We have focused here on inferred gaps, where it is uncertain whether they correspond to real events is different. In this case, a necessary step is to disentangle what fraction of the apparent signal results from the guide-tree and identify those informative gaps that are carrying truly new phylogenetic signal to avoid biases. As we have shown, in current algorithms, the guide-tree and arbitrary gap parameters seem to dominate the nature and strength of the signal carried by gaps. This effect may be even stronger in alignments with more sequences and higher divergence. We want to stress that our proposed measure of guide-tree dependency (i.e. *guidescore*) is not informative of the quality of the resulting alignment. A method highly dependent of the guide-tree may result in accurate or inaccurate alignments given the correct guide-tree. Alignment accuracy, mostly measured in terms of correct residue pairs, has been assessed in many previous studies (Kemena and Notredame, 2009) and has not been the purpose of this work. Our analyses on simulated sequences provide some insights into the difficulty of inserting gaps at correct positions, even when the correct guide-tree is used. Needless to say, our results concern a limited dataset, and it is unclear how these results extrapolate to datasets of different complexity (more or less sequences of more or less divergence, for instance). Future studies will certainly improve our understanding of accuracy of gap inference and guide-tree dependency in broader contexts.

Finally, we have shown possible solutions to alleviate the effects of a strong guide-tree dependency, which include iterative alignment reconstruction, use of consistency across different alignments and trimming.

## Supplementary Material

Supplementary Data
