# Supplementary material for: Measuring guide-tree dependency of inferred gaps in progressive aligners
Source: Bioinformatics. 2013 Feb 23;29(8):1011–7. doi: 10.1093/bioinformatics/btt095 (PMC3624810; doi:10.1093/bioinformatics/btt095)
Supplement: Supplementary Data [file supp_29_8_1011__index.html]

Measuring guide-tree dependency of inferred gaps in progressive aligners — Measuring guide-tree dependency of inferred gaps in progressive aligners — Supplementary Data 

# Measuring guide-tree dependency of inferred gaps in progressive aligners

## Supplementary Data

files

**Files in this Data Supplement:**

- Supplementary Data - pdf file
