# Supplementary material for: Measuring guide-tree dependency of inferred gaps in progressive aligners
Source: Bioinformatics. 2013 Feb 23;29(8):1011–7. doi: 10.1093/bioinformatics/btt095 (PMC3624810; doi:10.1093/bioinformatics/btt095)
Supplement: Supplementary Data [file supp_btt095_Capella_Gabaldon_Bioinformatics_supplementary.pdf]

Supplementary material for the article:

“Measuring guide-tree dependency of inferred gaps in progressive aligners”

Salvador Capella-Gutierrez and Toni Gabaldón*

Content:

1. Supplementary methods.
2. Supplementary figures and tables legends.
3. Literature cited.
4. Supplementary figures.
5. Supplementary tables.

1. Supplementary methods.

Simulated dataset.

The simulated dataset, which was used previously in (Capella-Gutierrez *et al*., 2009), contains 600 sets of 32 sequences divided in 6 categories according to the reference tree topology, asymmetric or symmetric, used to generate these artificial sequences and the level of divergence among the leaves in the reference trees, 0.5x, 1.0x and 2.0x. Reference trees were created after extending original trees published in (Talavera and Castresana, 2007) using ETE (Huerta-Cepas *et al*., 2011). These sets of evolutionary simulations of sequences were performed using ROSE v1.3 (Stoye *et al*, 1998) along the mentioned reference trees. The simulations included insertions and deletions with a probability of 0.03. The other parameters, including the seed protein, were used as described in (Talavera and Castresana, 2007). Shortly, patterns of rate heterogeneity were extracted from alignments of NAD2 orthologous sequences using the program TreePuzzle (Strimmer and von Haeseler, 1996) with a model of among-site rate heterogeneity that assumed a gamma distribution of rates. On this way, it is possible to simulate different levels of conservation along the sequence. The original profile contains 386 positions which was concatenated 1, 2, 3, 4 and 8 times to create sequences of different lengths. In our case, we only used sequences with, in average, 800 residues. The PAM evolutionary model [Dayhoff *et al*., 1978] was used to simulate the evolution of amino acids. Finally, a vector of the form [0.5, 0.4, 0.3, 0.2, 0.1] was used to simulated the relative frequency of indels with lengths from 1 to 5 residues, respectively.

2. Supplementary figures and tables legends.

Figure S1.

Graphical description about how to compute the precision and the accuracy for the same sequence in two alignments using one of them as reference. Alignment accuracy and precision are computed averaging individual sequences values.

Figure S2.

Canonical species trees for the different datasets used in the study. Sfig 1A shows the generic topology of (Dessimoz and Gil, 2010) for the different taxonomic clades (see below clades/species organization). Sfig 1B shows the species trees inferred by (Marcet-Houben and Gabaldón, 2009) corresponding to 12 *Saccharomycotina* species. Sfig 1C and Sfig 1D shows the topology used in (Capella-Gutierrez, et al., 2009) to simulate alignments containing 32 sequences. Branch lengths and scale have been intentionally ignored because the idea is to show the canonical topology of the different datasets.

Taxonomic clades and group for (Dessimoz and Gil, 2010).

Eukaryota:

G1. *Homo sapiens*: HUMAN

G2. *Other Primates:* MACMU, MICMU, OTOGA, PANTR, PONPA

G3. *Other Mammalia:* BOVIN, CANFA, CAVPO, DASNO, ECHTE, ERIEU, FELCA, HORSE, LOXAF, MONDO, MOUSE, MYOLU, OCHPR, ORNAN, RABIT, RATNO, SORAR, SPETR, TUPGB

G4. *Other Vertebrata:* CHICK, DANRE, FUGRU, GASAC, ORYLA, TETNG, XENTR

G5. Protostomia: AEDAE, ANOGA, APIME, DAPPU, DROME, DROPS, HELRO, LOTGI

G6. *Fungi:* ASHGO, ASPFU, BOTFB, CANAL, CANGA, CRYNE, DEBHA, ENCCU, KLULA, LODEL, MAGGR, PHANO, PICST, SCHPO, USTMA, YARLI, YEAST

Fungi:

G1. CANGA, YEAST

G2. ASHGO, KLULA

G3. CANAL, DEBHA

G4. YARLI

G5. ASPFU, SCHPO

G6. CRYNE, ENCCU

Bacteria:

G1. *Gammaproteobacteria*: ACIAD, ACIBT, ACTP2, ACTSZ, AERHH, AERS4, ALCBS, ALHEH, BAUCH, BLOFL, BLOPB, BUCAI, BUCAP, BUCBP, BUCCC, CARRP, CHRSD, COLP3, COXBU, DICNV, ECO24, ECO57, ECODH, ECOK1, ECOL5, ECOL6, ECOLI, ECOUT, ENT38, ERWCT, FRAT1, FRATF, FRATH, FRATN, FRATO, FRATT, FRATW, HAEDU, HAEI8, HAEIE, HAEIG, HAEIN, HAES1, HAHCH, HALHL, IDILO, KLEP7, LEGPA, LEGPC, LEGPH, LEGPL, MANSM, MARAV, MARMS, METCA, NITOC, PASMU, PHOLL, PHOPR, PSE14, PSEA6, PSEA7, PSEAB, PSEAE, PSEE4, PSEF5, PSEHT, PSEMY, PSEP1, PSEPF, PSEPG, PSEPK, PSEPW, PSESM, PSEU2, PSEU5, PSYAR, PSYCK, PSYIN, PSYWF, RUTMC, SACD2, SALAR, SALCH, SALPA, SALTI, SALTY, SERP5, SHEAM, SHEB5, SHEB8, SHEB9, SHEDO, SHEFN, SHEHH, SHELP, SHEON, SHEPA, SHEPC, SHESA, SHESH, SHESM, SHESR, SHESW, SHIBS, SHIDS, SHIF8, SHIFL, SHISS, SODGM, STRMK, THICR, VESOH, VIBCH, VIBF1, VIBPA, VIBVU, VIBVY, WIGBR, XANAC, XANC5, XANC8, XANCB, XANCP, XANOM, XANOR, XYLFA, XYLFT, YERE8, YERP3, YERPA, YERPE, YERPN, YERPP, YERPS

G2. *Betaproteobacteria*: ACIAC, ACISJ, AZOSB, AZOSE, BORA1, BORBR, BORPA, BORPD, BORPE, BURCA, BURCH, BURCM, BURM7, BURMA, BURP0, BURP1, BURP6, BURPS, BURS3, BURTA, BURVG, BURXL, CHRVO, DECAR, HERAR, JANMA, METFK, METPP, NEIG1, NEIM0, NEIMA, NEIMB, NEIMF, NITEC, NITEU, NITMU, POLNA, POLSJ, POLSQ, RALEH, RALEJ, RALME, RALSO, RHOFD, THIDA, VEREI

G3. *Alphaproteobacteria*: AGRT5, ANAMM, ANAPZ, AZOC5, BARBK, BARHE, BARQU, BRAJA, BRASB, BRASO, BRUA2, BRUAB, BRUME, BRUO2, BRUSI, BRUSU, CAUCR, EHRCJ, EHRCR, EHRRG, EHRRW, ERYLH, GLUDA, GLUOX, GRABC, HYPNA, JANSC, MAGMM, MARMM, MESSB, NEOSM, NITHX, NITWN, NOVAD, OCHA4, ORITB, PARDP, PARL1, PELUB, RHIEC, RHIL3, RHILO, RHIME, RHOP2, RHOP5, RHOPA, RHOPB, RHOPS, RHORT, RHOS1, RHOS4, RHOS5, RICAH, RICB8, RICBR, RICCK, RICCN, RICFE, RICM5, RICPR, RICRO, RICRS, RICTY,ROSDO, SILPO, SILST, SINMW, SPHAL, SPHWW, WOLPM, WOLTR, ZYMMO

G4. *Deltaproteobacteria*: ANADE, ANADF, BDEBA, DESDG, DESPS, DESVH, DESVV, GEOMG, GEOSL, GEOUR, LAWIP, MYXXD, PELCD, PELPD, SORC5, SYNAS, SYNFM *and Epsilonproteobacteria*: ARCB4, CAMC1, CAMC5, CAMFF, CAMJ8, CAMJD, CAMJE, CAMJJ, CAMJR, HELAH, HELHP, HELPH, HELPJ, HELPY, NITSB, SULDN, SULNB, WOLSU

G5. *Spirochaetes*: BORAP, BORBU, BORGA, LEPBJ, LEPBL, LEPIC, LEPIN, TREDE, TREPA

G6. *Firmicutes*: ACHLI, ALKMQ, ALKOO, AYWBP, BACA2, BACAH, BACAN, BACC1, BACCN, BACCR, BACCZ, BACHD, BACHK, BACLD, BACSK, BACSU, BACWK, CARHZ, CLOAB, CLOB1, CLOB8, CLOBH, CLOBK, CLOD6, CLOK5, CLONN, CLOP1, CLOPE, CLOPH, CLOPS, CLOTE, CLOTH, DESHY, ENTFA, EXIS2, GEOKA, GEOTN, LACAC, LACBA, LACC3, LACDA, LACDB, LACGA, LACH4, LACJO, LACLA, LACLM, LACLS, LACPL, LACRF, LACS1, LACSS, LEUMM, LISIN, LISMF, LISMO, LISW6, MESFL, MOOTA, MYCCT, MYCGA, MYCGE, MYCH2, MYCH7, MYCHJ, MYCMO, MYCMS, MYCPE, MYCPN, MYCPU, MYCS5, NATTJ, OCEIH, OENOB, ONYPE, PEDPA, STAA1, STAA2, STAA3, STAA8, STAA9, STAAB, STAAC, STAAE, STAAM, STAAN, STAAR, STAAS, STAAT, STAAW, STAEQ, STAES, STAHJ, STAS1, STRA1, STRA3, STRA5, STRGC, STRMU, STRP1, STRP2, STRP3, STRP6, STRP8, STRPB, STRPC, STRPD, STRPF, STRPG, STRPM, STRPN, STRR6, STRS2, STRSV, STRSY, STRT1, STRT2, STRTD, SYNWW, THETN, UREPA

Figure S3

Mean distance, in term of wrong splits, to the Canonical tree of Gap parsimony trees after forcing the use of 100 different guide-trees with the maximum split-distance to the reference tree.

Figure S4

Alternative trees with maximum split-distance trees to the Canonical trees shown in Figure S1. The ETE package (Huerta-Cepas, et al., 2010) was used to perform all operations related to phylogenetic trees.

Figure S5

Similar to figure 1 in the main text, it shows mean distance, in term of wrong splits, to the

wrong tree, tree with maximum split-distance to the canonical one, of the different Gap parsimony trees reconstructed after allowing to the programs to build its own guide-tree (blue dots) or forcing to use either the canonical tree (green diamonds) or the wrong tree (red squares). Wrong splits measure the number of topological differences between two given trees.

Figure S6.

It includes the comparison, in term of wrong splits, of the guide-tree inferred by the programs (yellow dots) as first step to make the Multiple Sequence Alignments to the Canonical trees. The figure includes as well all points showed in figure 1 in the main text.

Figure S7.

Similar to figure 3 in the main text, this plot shows the *guidescore* values for different alignment programs in the context of 2 real and 1 simulated datasets. To compute these values, two randomly generated trees with the highest topological distance between them were used instead of using a canonical and “wrong tree”.

Figure S8.

This plot shows the effect of varying the topological distance of guide-trees over the *guidescore* values. To generate this plot, a set of guide-trees were used to reconstruct the alignments ranging from the closest one, in terms of topological distance (Robinson&Foulds distance = 2), to the canonical tree to the most distant one (Robinson&Foulds distance = 6). The plot contains 3 subplots which correspond to the 3 datasets: Bacteria (top panel), Eukaryota (central panel) and Fungi (bottom panel) published originally in (Dessimoz and Gil, 2010).

Figure S9.

Similar to figure S6, this plot shows the result of varying progressively one of the trees used to compute the *guidescore* values from a very similar topology (distance = 2 in terms of Robinson&Fould distance) to a very different one (distance = 16 in terms of Robinson&Fould distance) compared with the canonical tree. The results have been generated in the context of the *yeast* dataset, originally published in (Marcet-Houben and Gabaldón, 2009).

Figure S10.

Similar to figure S6, this plot shows the result of varying progressively one of the trees used to compute the guidescore values from a very similar topology (distance = 2 in terms of Robinson&Fould distance) to a very different one (distance = 58 in terms of Robinson&Fould distance) compared to the canonical trees used for these datasets. The dataset is based on simulated sequences originally published in (Capella-Gutierrez et al., 2009).

Figure S11.

Similar to figure 3 in the main text, it shows the *guidescores* for three alternative approaches to alleviate the guide-tree dependence effect in the final alignment. New approaches includes are the iterative estimation at the same time of the alignment and the Maximum Likelihood tree done by SATe II (Liu, et al., 2012) either using the default alignment program: Mafft or using Prank+F, and the reconstruction of the Multiple Sequence Alignment after considering different input alignments done by M-Coffee (Wallace, et al., 2006).

Figure S12.

Similar to figure 1 in the main text, it shows the accuracy and precision,in terms of gaps placements, for the two approximations which co-estimate iteratively the tree and the alignment: SATé II and SATe II with Prank+F. Green and blue colors from the bars are used to distinguish the different nature of the simulated data, asymmetric and symmetric respectively. Light colors bars are used to show the results when using the normal procedure and the dark colors ones are used when the correct tree was used as input.

Table S1.

Percentage of wrong splits against the reference trees for the concatenation of individual presence/absence gap patterns alignments. Alignments were concatenated accordingly to the method used to infer them as well as to the corresponding dataset. Although, three main datasets have been used on this work, only two of them, one with real data (yeast) and the one with simulated data, were used. Regarding to the data originally published in (Dessimoz and Gil, 2010), concatenated alignments could not be generated since each case contains a specific set of species and therefore there is not a unique reference tree for each dataset in the benchmark.

3. Literature cited

Capella-Gutierrez, S., Silla-Martinez, J.M., Gabaldón, T. trimAl: a tool for automated alignment trimming in large-scale phylogenetic analyses. *Bioinformatics* 2009:25, 1972-1973.

Dayhoff, M. O., Schwartz, R.M., Orcutt B.C. (1978) A model of evolutionary change in proteins. pages 345−352 in Atlas of protein sequence structure (M. O. Dayhoff, ed.) National Biomedical Research Foundation, Washington, D.C

Dessimoz, C., Gil, M. Phylogenetic assessment of alignments reveals neglected tree signal in gaps, *Genome Biology* 2010:11,R37.

Huerta-Cepas, J., Dopazo, J. and Gabaldón, T. ETE: a python Environment for Tree Exploration, *BMC Bioinformatics* 2010:11, 24.

Marcet-Houben, M., Gabaldón, T. The tree versus the forest: the fungal tree of life and the topological diversity within the yeast phylome, *PLoS One* 2009:4,e4357.

Landan G, Graur D. Heads or tails: a simple reliability check for multiple sequence alignments. *Molecular biology and evolution*. 2007;24(6):1380-3.

Liu K, Warnow TJ, Holder MT, et al. SATe-II: very fast and accurate simultaneous estimation of multiple sequence alignments and phylogenetic trees. *Systematic biology*. 2012;61(1):90-106.

Stoye J, Evers D, Meyer F. Rose: generating sequence families. *Bioinformatics,* 1998;14(2):157-63.

Strimmer, K., von Haeseler, A. Quartet puzzling: A quartet maximum-likelihood method for reconstructing tree topologies. *Molecular biology and evolution.* 1996;13:964–969.

Thompson, J.D., Koehl, P., Ripp, R. and Poch, O. BAliBASE 3.0: latest developments of the multiple sequence alignment benchmark, *Proteins* 2005;61:127-136.

Wallace IM, O'Sullivan O, Higgins DG, Notredame C. M-Coffee: combining multiple sequence alignment methods with T-Coffee. *Nucleic acids research*. 2006;34(6): 1692-1699.

4. Supplementary figures.

Figure S1

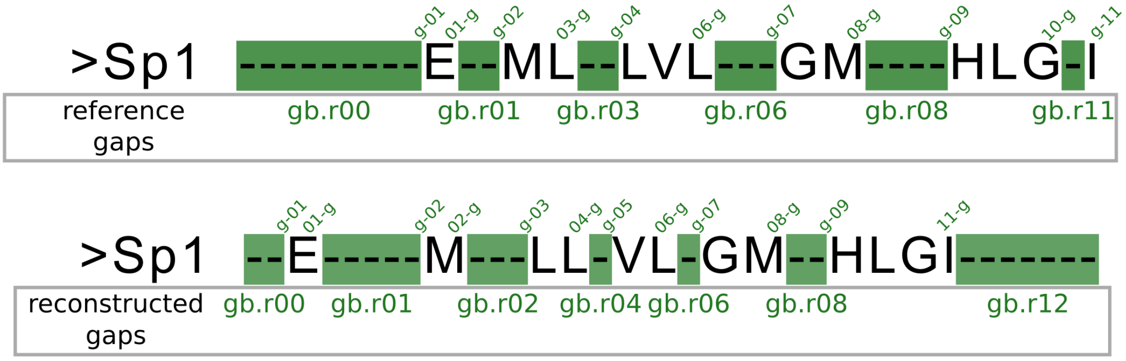

True Positives (TP) = len(reference gaps ∩ reconstructed gaps)

False Positives (FP) = len(reconstructed gaps - reference gaps)

True Negatives (TN) = len(reference residues ∩ reconstructed residues)*

False Negatives (FN) = len(reference gaps - reconstructed gaps)

Precision = TP / (TP + FP)

Accuracy = (TP + TN) / (TP + FP + TN + FN)

* Only residues next to a gap blocks

Figure S2

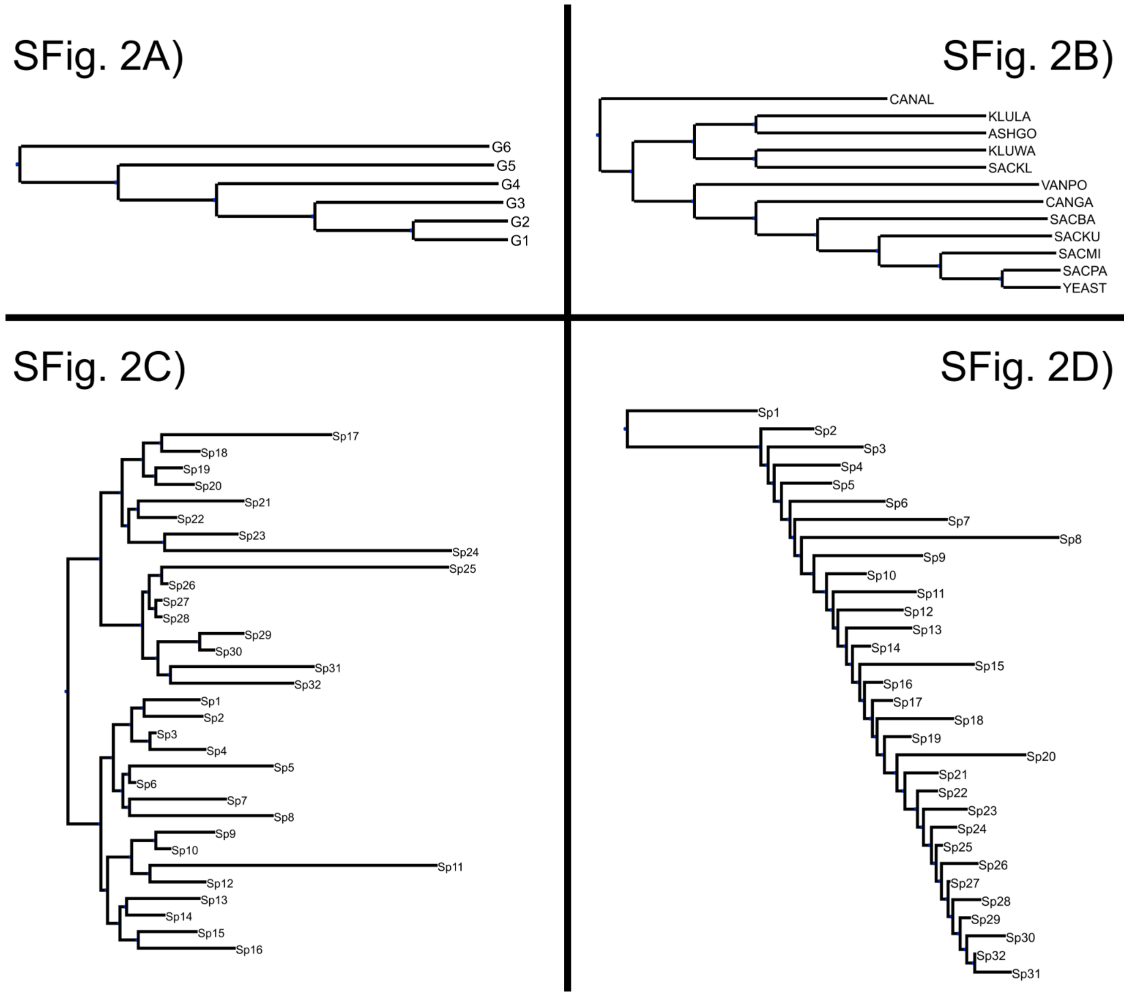

Figure S3

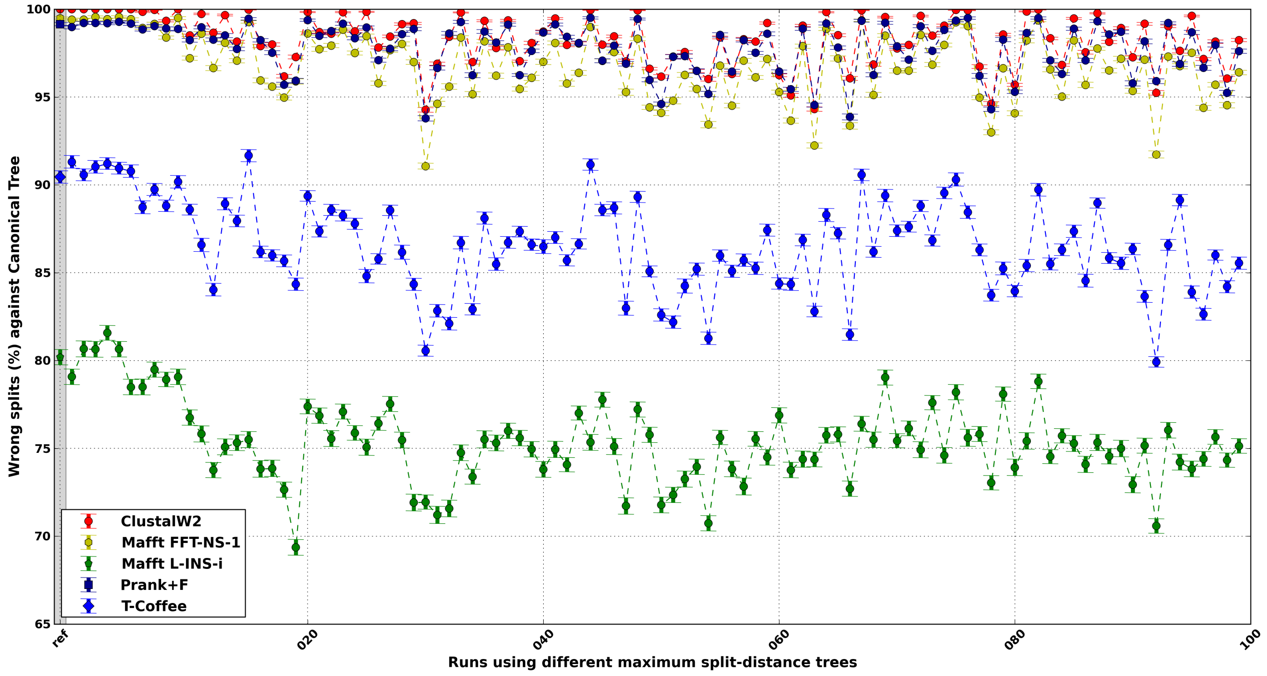

Figure S4

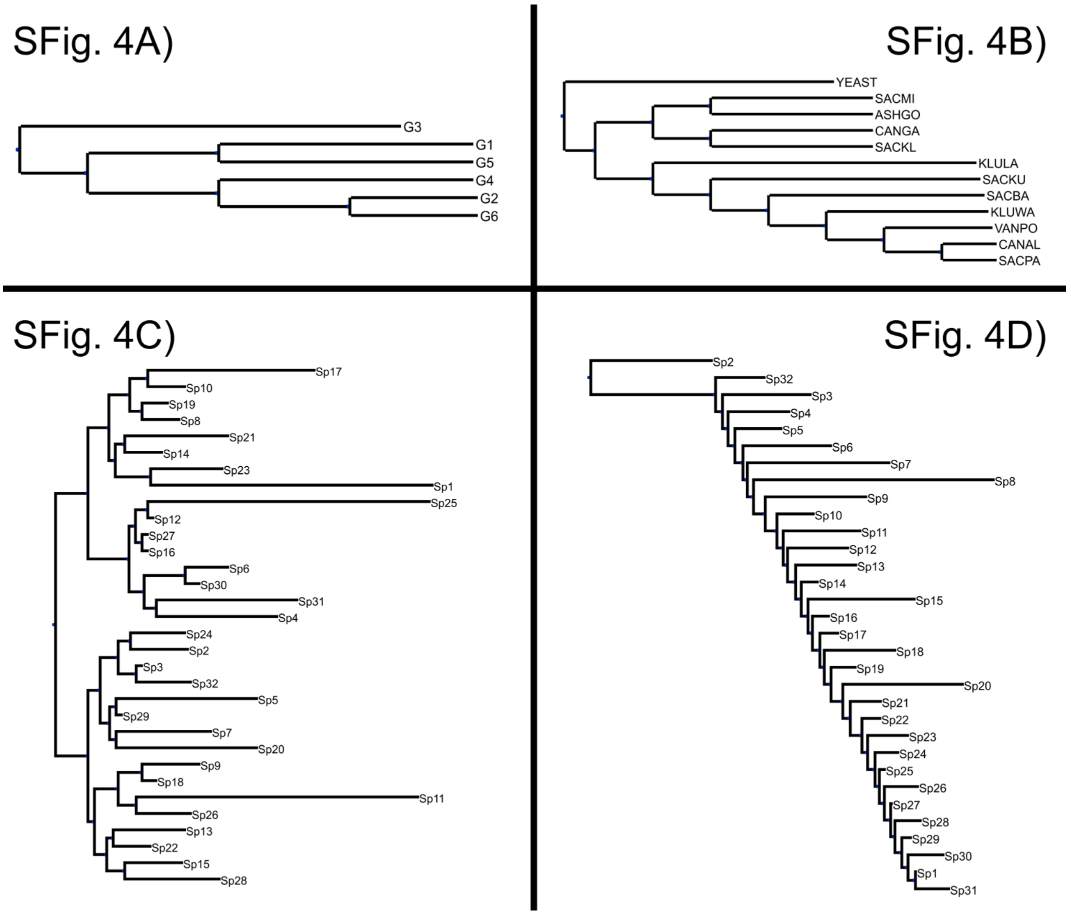

Figure S5

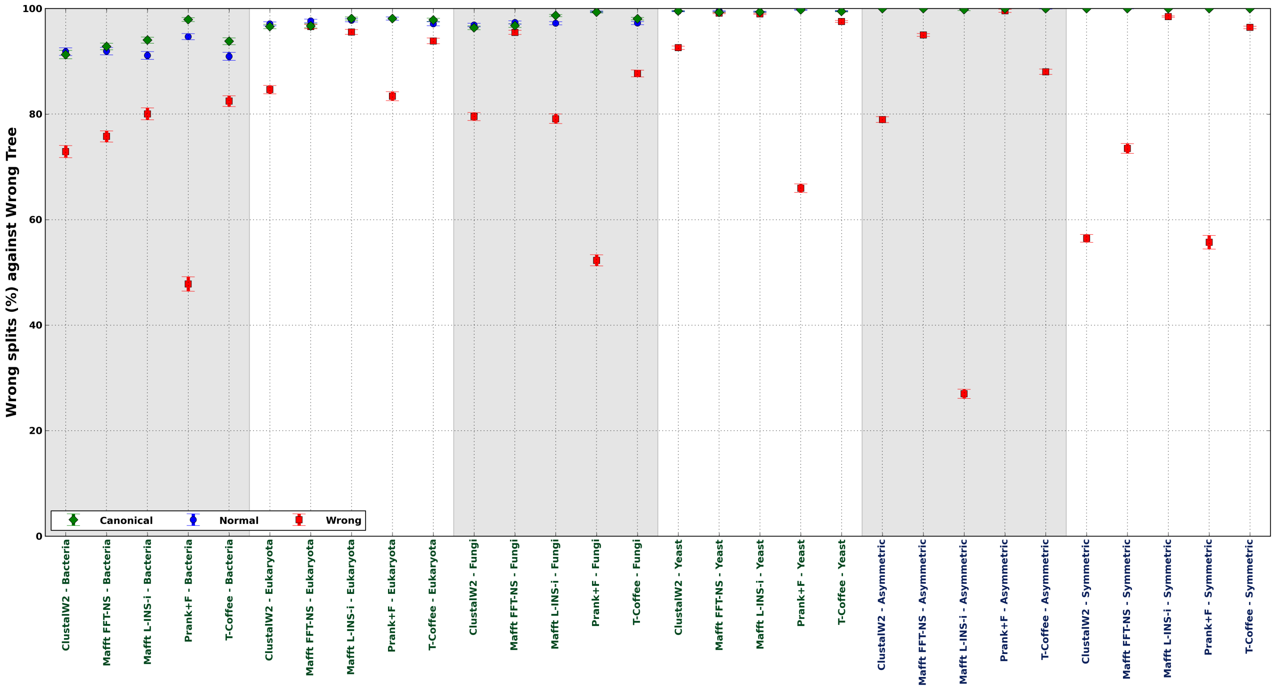

Figure S6

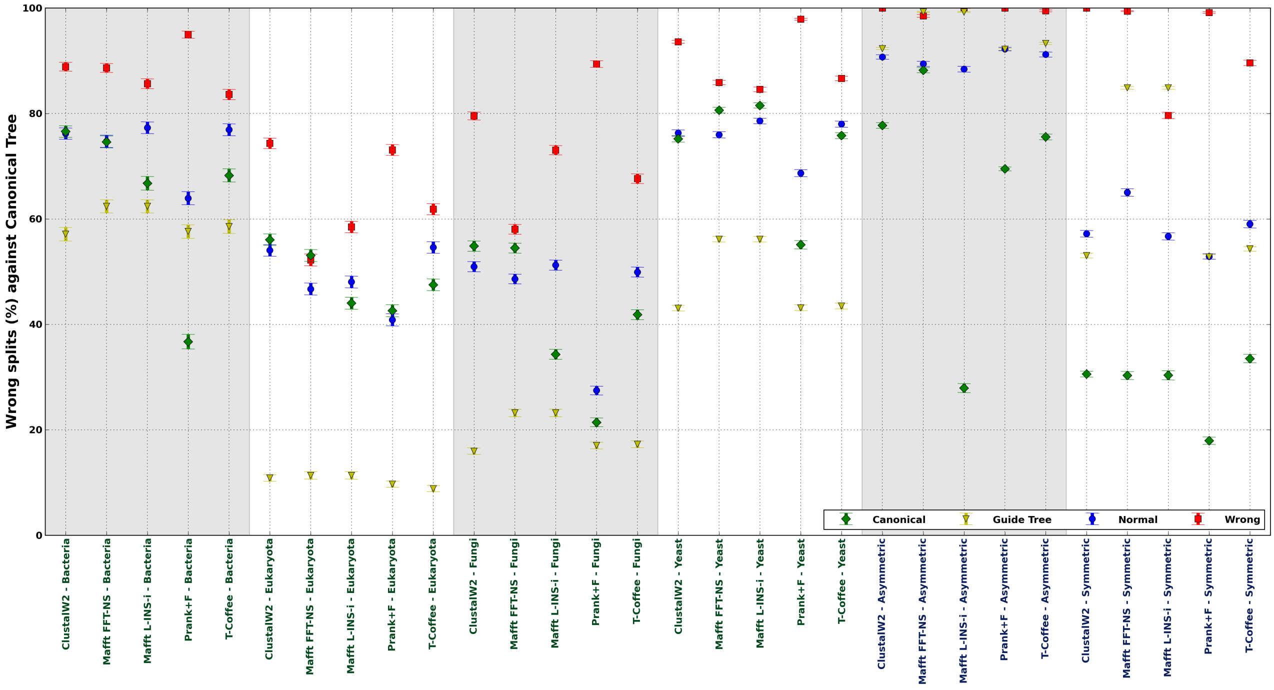

Figure S7

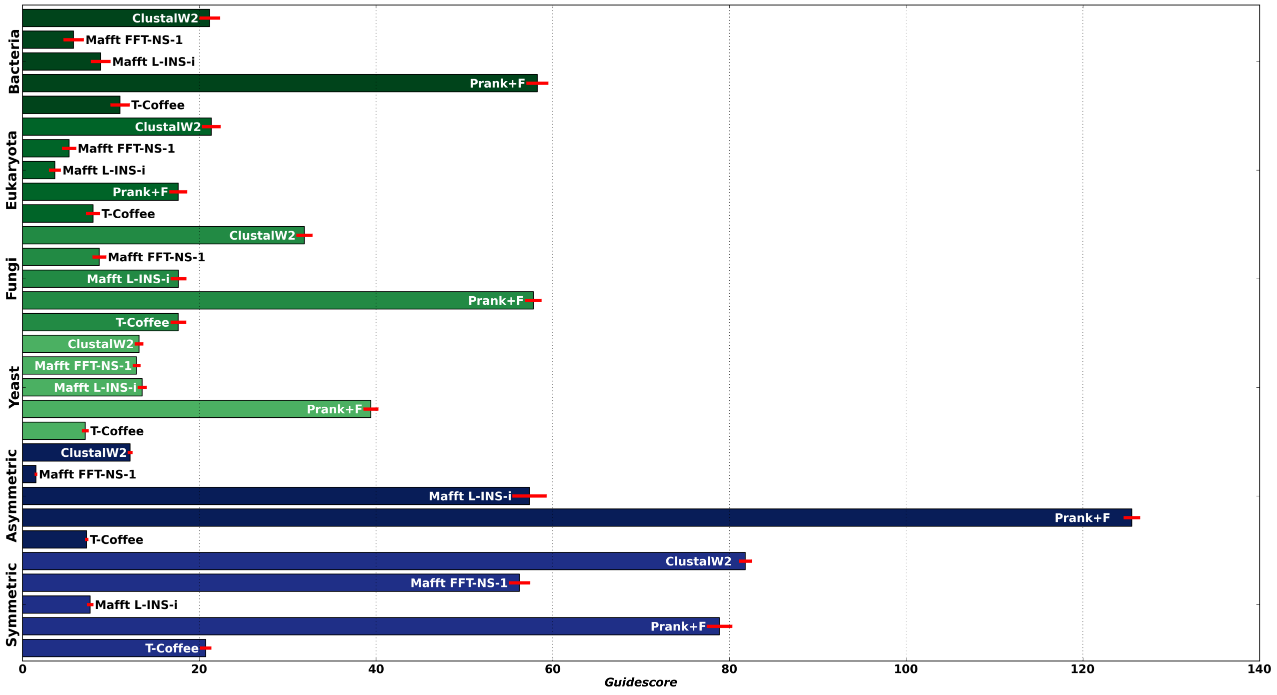

Figure S8

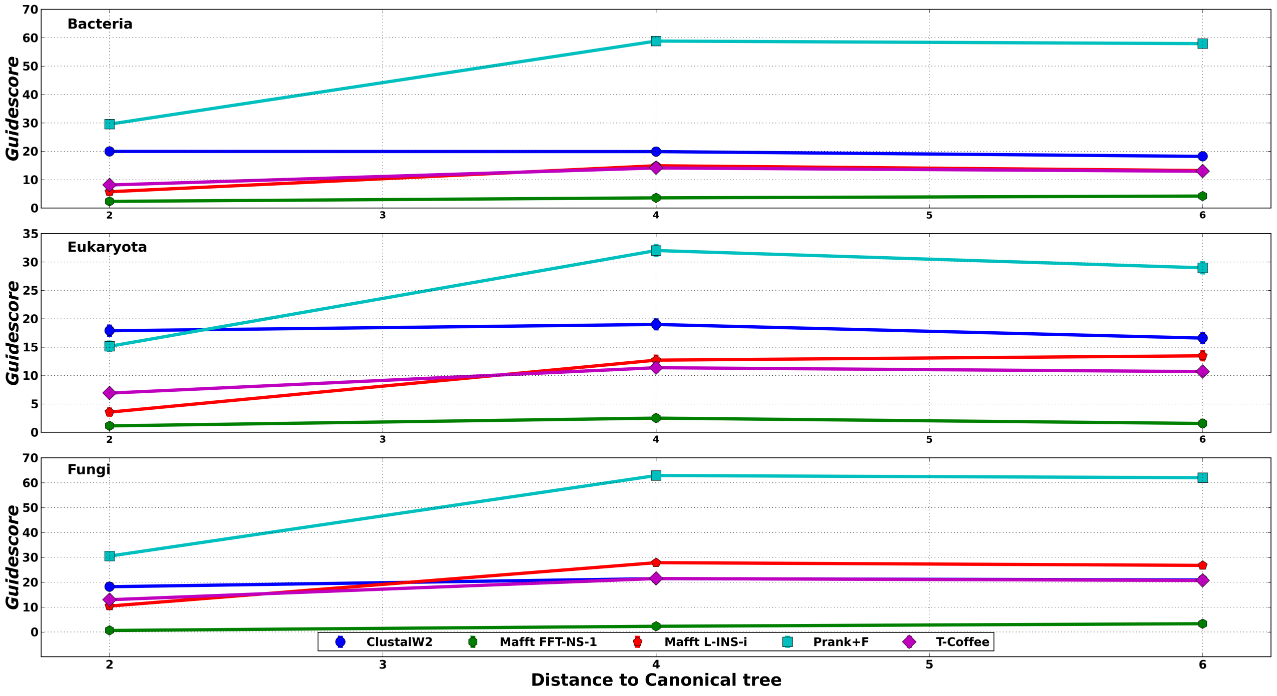

Figure S9

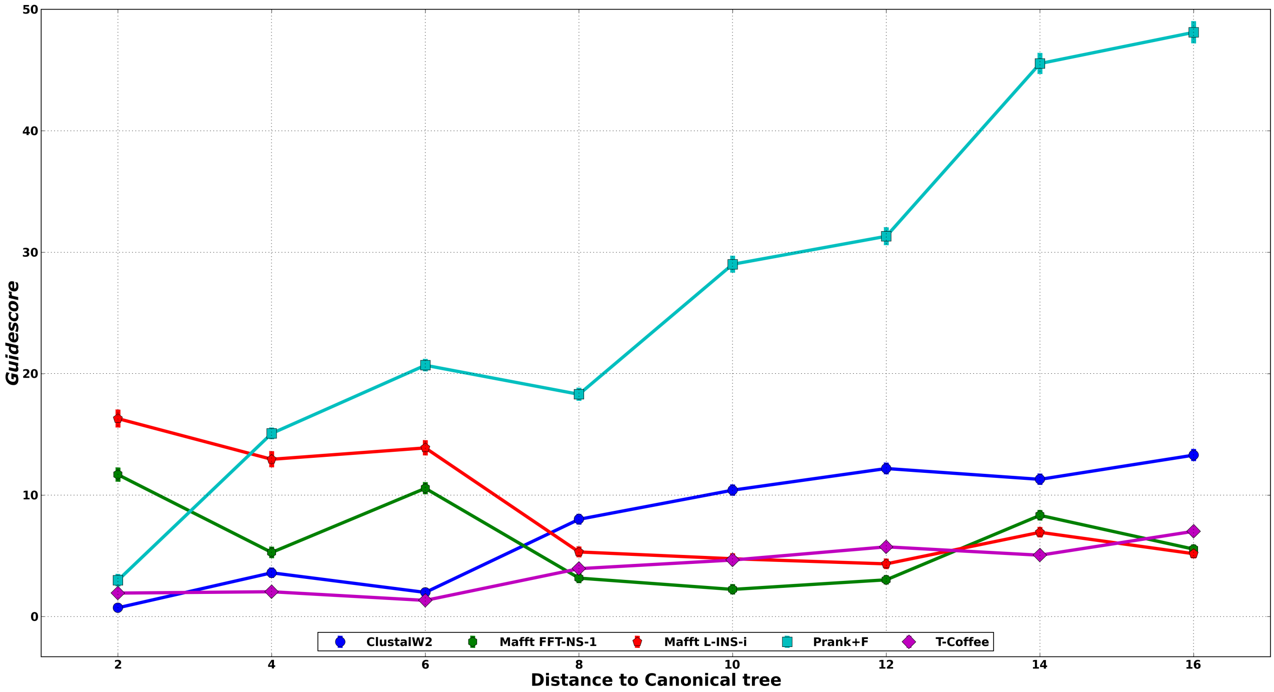

Figure S10

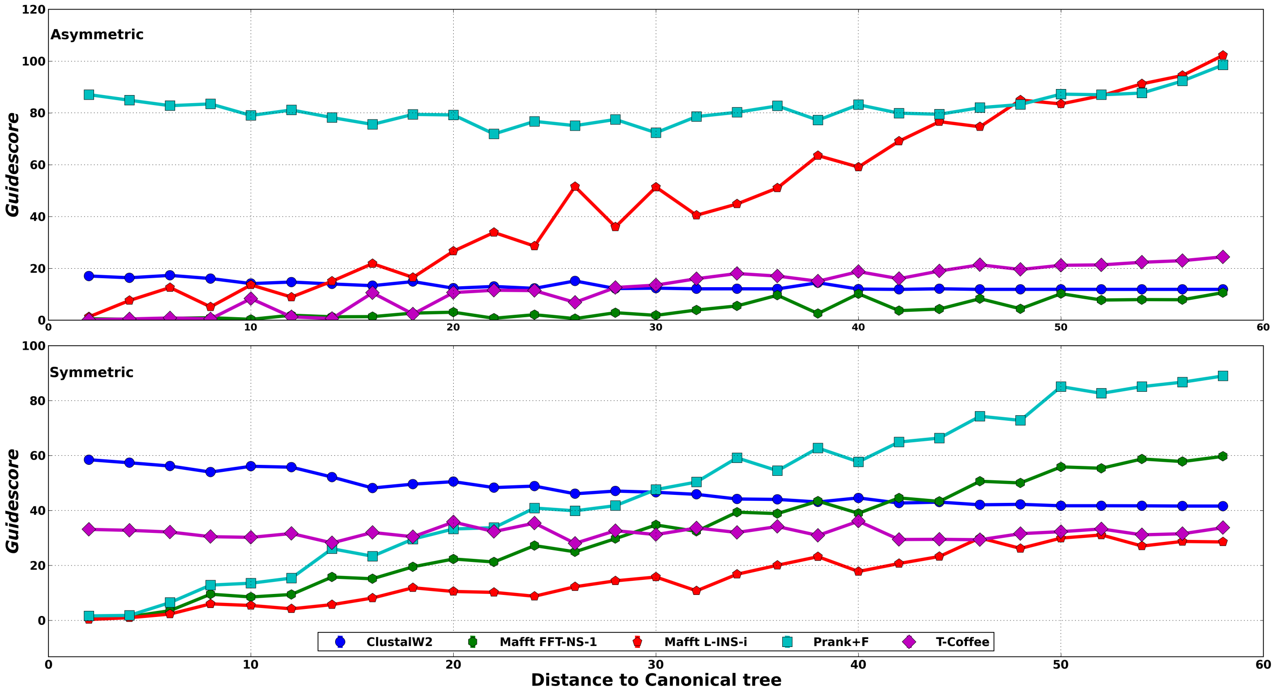

Figure S11

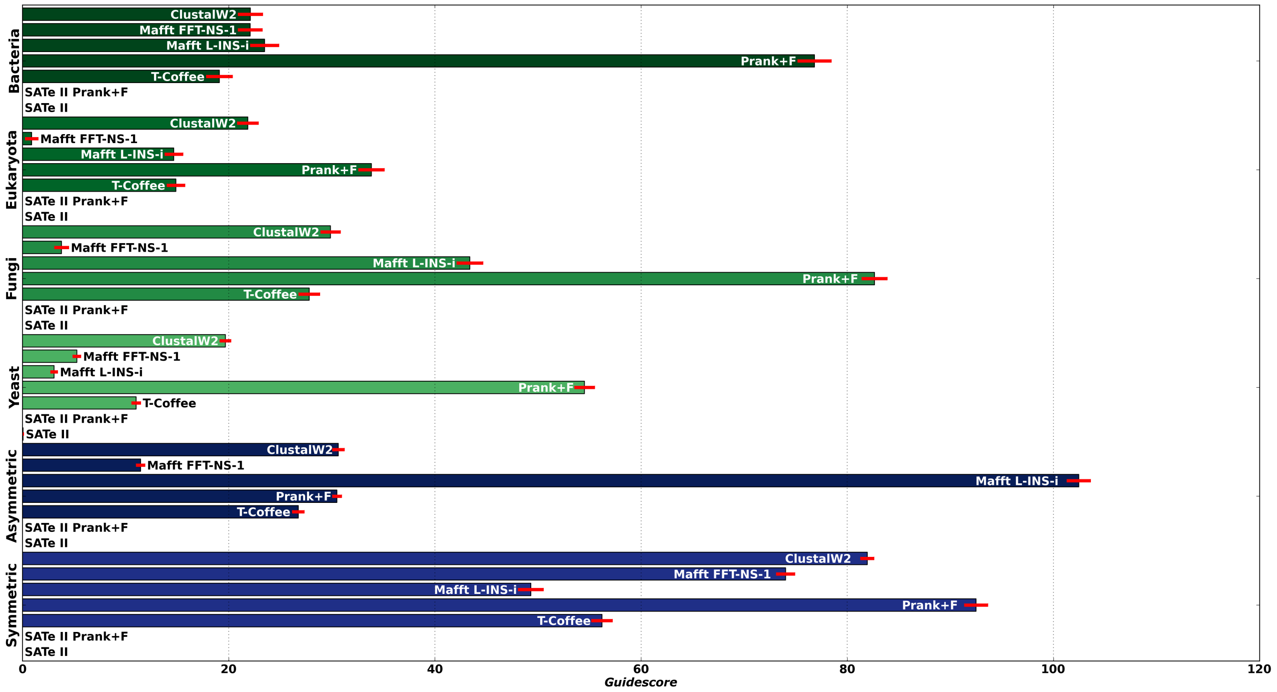

Figure S12

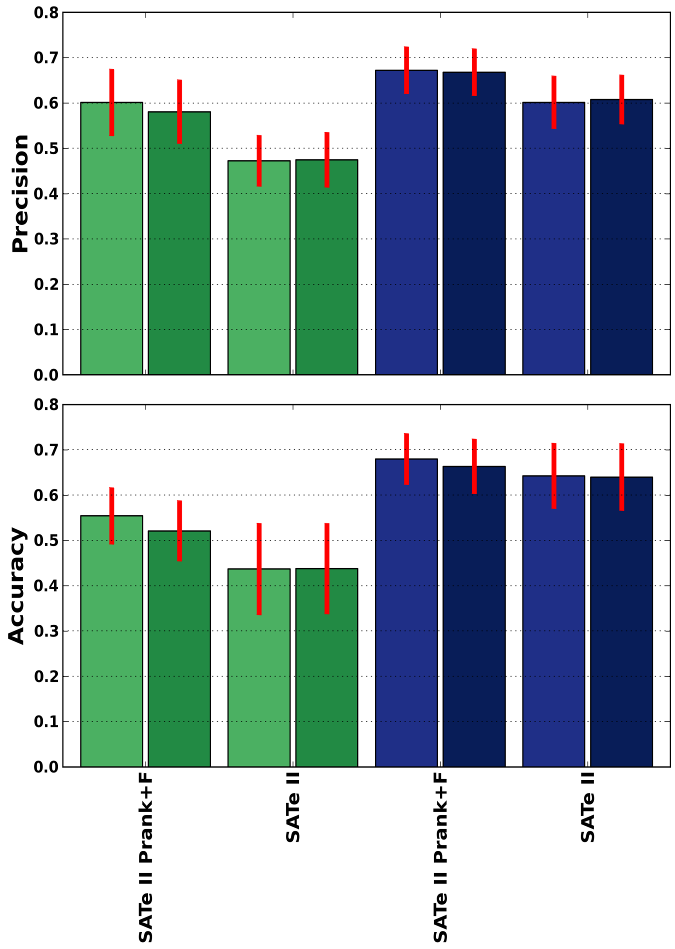

5. Supplementary tables.

Table S1

	Dataset	# Genes	Strategy / Program	Clustal W2	Mafft FFT-NS	Mafft L-INS-i	Prank+F	T-Coffee	SATé II	SATé II Prank+F
Simulated	Symmetric	300	Wrong	100%	100%	72%	100%	93%	10%	28%
			Normal	31%	55%	20%	38%	31%	10%	34%
			Canonical	0%	0%	0%	0%	0%	10%	28%
			Reference	0%						
	Asymmetric	300	Wrong	100%	100%	100%	100%	100%	62%	90%
			Normal	76%	76%	76%	86%	76%	62%	86%
			Canonical	38%	55%	0%	76%	38%	62%	76%
			Reference	41%						
Real	Yeast	857	Wrong	100%	55%	44%	100%	55%	22%	11%
			Normal	22%	11%	11%	22%	22%	11%	22%
			Canonical	11%	22%	33%	11%	22%	22%	22%
